# Different mechanisms of X-ray irradiation-induced male and female sterility in *Aedes aegypti*

**DOI:** 10.1186/s12915-023-01757-1

**Published:** 2023-11-27

**Authors:** Heng Zhang, Emma Trueman, Xinjun Hou, De Xian Chew, Lu Deng, Jonathan Liew, Tania Chia, Zhiyong Xi, Cheong Huat Tan, Yu Cai

**Affiliations:** 1 Temasek Life Sciences Laboratory, National University of Singapore, Singapore, 117604 Singapore; 2 Present address: Institute of Infectious Disease, Shenzhen Bay Laboratory, Shenzhen, 518000 China; 3 Department of Biological Sciences, National University of Singapore, Singapore, 117543 Singapore; 4 Environmental Health Institute, National Environment Agency, Singapore, 138667 Singapore; 5 Department of Microbiology and Molecular Genetics, Michigan State University, East Lansing, MI 48824 USA

**Keywords:** *Aedes aegypti*, Mosquito, Sterile insect technique, Incompatible insect technique, Germline, Germline stem cells, X-ray irradiation, Ovary, Testis, *Wolbachia*

## Abstract

**Background:**

*Aedes aegypti* (*Ae. aegypti*) is the major vector that transmits many diseases including dengue, Zika, and filariasis in tropical and subtropical regions. Due to the growing resistance to chemical-based insecticides, biological control methods have become an emerging direction to control mosquito populations. The sterile insect technique (SIT) deploys high doses of ionizing radiation to sterilize male mosquitoes before the release. The *Wolbachia*-based population suppression method of the incompatible insect technique (IIT) involves the release of *Wolbachia*-infected males to sterilize uninfected field females. Due to the lack of perfect sex separation tools, a low percentage of female contamination is detected in the male population. To prevent the unintentional release of these *Wolbachia*-infected females which might result in population replacement, a low dose of X-ray irradiation is deployed to sterilize any female escapees. However, it remains unclear whether these irradiation-induced male and female sterilizations share common mechanisms.

**Results:**

In this work, we set out to define the minimum dose of X-ray radiation required for complete female sterilization in *Ae. aegypti* (NEA-EHI strain). Further results showed that this minimum dose of X-ray irradiation for female sterilization significantly reduced male fertility. Similar results have been reported previously in several operational trials. By addressing the underlying causes of the sterility, our results showed that male sterility is likely due to chromosomal damage in the germ cells induced by irradiation. In contrast, female sterility appears to differ and is likely initiated by the elimination of the somatic supporting cells, which results in the blockage of the ovariole maturation. Building upon these findings, we identified the minimum dose of X-ray irradiation on the *Wolbachia*-infected NEA-EHI (*w*AlbB-SG) strain, which is currently being used in the IIT-SIT field trial. Compared to the uninfected parental strain, a lower irradiation dose could fully sterilize *w*AlbB-SG females. This suggests that *Wolbachia*-carrying mosquitoes are more sensitive to irradiation, consistent with a previous report showing that a lower irradiation dose fully sterilized *Wolbachia*-infected *Ae. aegypti* females (Brazil and Mexican strains) compared to those uninfected controls.

**Conclusions:**

Our findings thus reveal the distinct mechanisms of ionizing X-ray irradiation-induced male or female sterility in *Ae. aegypti* mosquitoes, which may help the design of X-ray irradiation-based vector control methods.

**Supplementary Information:**

The online version contains supplementary material available at 10.1186/s12915-023-01757-1.

## Background

Globally, an estimated 17% of all infectious diseases are vector-borne diseases [1]. One of the most prolific vectors, *Ae. aegypti*, transmits many diseases including dengue, Zika, and yellow fever in the tropics and subtropics [2]. The prolific nature of *Ae. aegypti*, being influenced by the changing climate environments and increasing global trade, has put an estimated 3 billion people at risk of dengue worldwide [3]. There has been a concerning four-fold increase in dengue incidence in the last 50 years, which, coupled with the growing prevalence of worldwide insecticide resistance among *Aedes* species, has led to major calls for the innovation and utilization of more modern, sustainable, and complementary forms of vector control [4–8].

The SIT is a highly species-specific biological control method that involves the mass release of irradiation-sterilized male mosquitoes to mate with the field females, resulting in the production of non-viable eggs by these females [9–11]. Continuous release of sterile males will lead to the suppression of field mosquitoes in the target region. In SIT programmes, the underlying mechanism of sterilization is believed to be the chromosomal damage induced by the breaking of molecular bonds, the creation of free ions, and the formation of free radicals [12, 13]. In general, irradiation has a stronger deleterious effect on proliferating cells than post-mitotic cells. Spermatogenesis initiates at the anterior tip of the testis where germline stem cells (GSCs) undergo successive mitosis and meiosis, and progress through spermatogonia, spermatocyte, and spermatid to produce spermatozoa [13]. Earlier stages of spermatogenesis, especially the mitotic spermatogonia and meiotic spermatocytes, are more sensitive to radiation and are more likely to experience irreversible irradiation-induced chromosomal damage and are eliminated [14]. Irradiation on spermatids and spermatozoa could also lead to dominant lethal mutations. These dominant lethal mutations are randomly generated upon irradiation but do not interfere with the maturation of the gamete; instead, they are passed on to the embryo upon fertilization [12]. The damage from these dominant lethal mutations is manifested during zygote development, leading to the accumulation of severe chromosomal imbalances that ultimately result in embryonic death [12]. In addition to germ cells, mitotically active somatic cells are also damaged by irradiation [15]. Cell damage can be manifested by a decrease in performance traits such as reduced longevity in the irradiated mosquitoes—one of the major disadvantages of the SIT programme [12]. The higher the irradiation dose applied, the more severe the damage to the insect cells. Thus, it is important to understand the relationship between radiation dose and insect performance traits [12].

Many SIT-based mosquito control programmes have been conducted, with initial pilot trials performed in the 1960s and 1970s against *Culex quinquefaciatus* and *Anopheles quadrimaculatus* in Florida, USA, and *Anopheles albimanus* in El Salvador [15, 16]. Other small-scale pilot trials have been conducted on isolated islands or villages and have revealed the impact on entomological indicators such as the number of eggs collected from ovitraps, egg hatch rate, and adult catch rate [16]. Another SIT trial was conducted in northern Italy between 2005 and 2009 with a reduction in *Ae. albopictus* population occurring where release ratios were high, showing the possibility of mosquito control in small (10–17 ha) urban release sites [12, 17].

In addition to the renewed interest in SIT-based mosquito control in recent years, the incompatible insect technique (IIT) is another promising vector control tool [18, 19]. IIT involves the release of male mosquitoes infected with the endosymbiont bacterium, *Wolbachia*, and its ability to induce the biological mechanism of cytoplasmic incompatibility (CI) in female mosquitoes [18–20]. CI occurs when male mosquitoes infected with *Wolbachia* mate with female mosquitoes that are *Wolbachia*-free or infected with another strain of *Wolbachia* resulting in unviable eggs due to failure in early embryogenesis [21, 22]. *Wolbachia* and its ability to induce CI have been explored for mosquito population suppression for many decades [18, 23].

The IIT-based population suppression approach has been shown to be a promising tool for suppressing mosquito vector populations [24–26]. However, due to the absence of a perfect method for sex separation and imperfect maternal transmission, the combination of SIT and IIT has been implemented in several programmes [24–27]. While high doses of ionizing irradiation are employed in the SIT to achieve near-complete (> 99%) male sterility, a lower dose of irradiation is used in the IIT-SIT approach to sterilize any female pupae that slip through the sex separation process [13, 16, 28, 29]. The combined IIT-SIT strategy creates a mutually beneficial system that aims for population suppression while eliminating the risk of population replacement by ensuring any *Wolbachia*-infected females that may be inadvertently released together with the males are infertile [29]. Furthermore, the risk of pathogen transmission by the *Wolbachia*-infected female mosquitoes is also significantly lower when compared to wild-type mosquitoes due to the pathogen-blocking activity of the *Wolbachia* [30–34]. The combined IIT-SIT strategy has been demonstrated successfully to suppress *Ae. albopictus* population in Guangzhou, China, and *Ae. aegypti* population in Thailand, Singapore and Mexico [24–27]. IIT-SIT is hence an effective and environmentally friendly strategy for mosquito population suppression and reducing disease transmission. With this success, the mechanism, however, is not fully understood and there is a need to understand the mechanism of X-ray radiation-induced male and female sterility in *Ae. aegypti*.

In this study, we set out to define the parameters for X-ray irradiation in *Ae. aegypti* (NEA-EHI strain) by addressing the minimum radiation dose for complete female sterilization with minimal effect on males under laboratory conditions. We also investigated its effect on mosquito’s performance traits by examining factors such as longevity, fecundity, fertility, and exploring the underlying mechanisms of female and male sterility. Based on these, we further examined the dose of X-ray radiation for female sterilization of *Wolbachia*-infected *Ae. aegypti* (*w*AlbB-SG strain) used in the IIT-SIT approach under laboratory conditions.

## Results

### Defining X-ray radiation dose for female sterilization

**Fig. 1:**
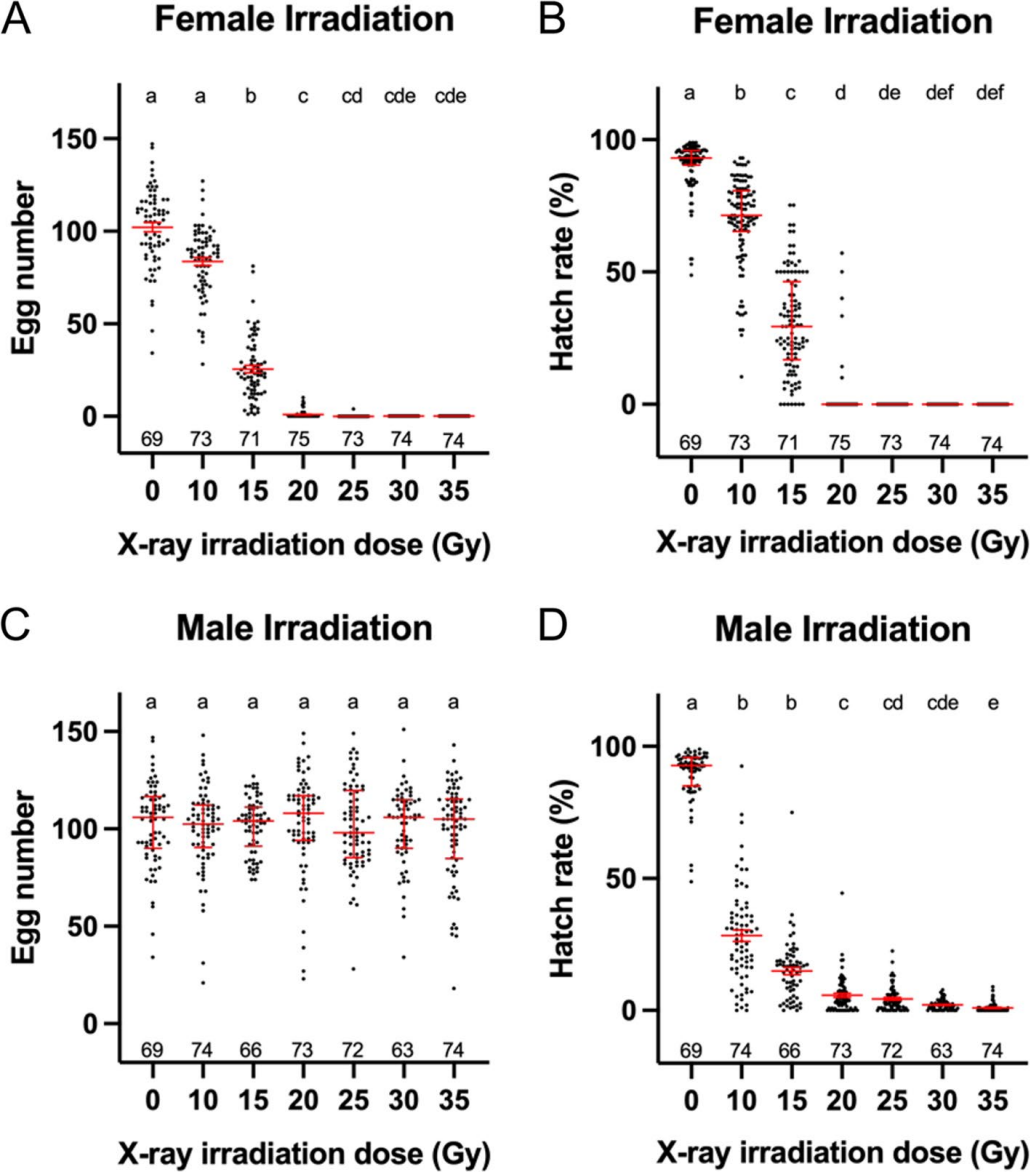
Fecundity and fertility after various X-ray irradiation doses. Effects of various doses of X-ray irradiation on egg production (**A**) and hatch rate (**B**) in irradiated females mated with wild-type males. Effects of various doses of X-ray irradiation on egg production (**C**) and hatch rate (**D**) in wild-type females mated with irradiated males. The number of samples in each group is shown above the X-axis. Error bars represent the median and interquartile range. Kruskal-Wallis test and Dunn’s multiple comparisons post hoc test shows the difference between control and individual irradiation dose; doses not sharing the same letter are significantly different

To determine an optimal X-ray dose for use in the operational tests, we first tested the lowest dose that could completely sterilize *Ae. aegypti* females and have a minimum impact on male mosquitoes under laboratory conditions. To this end, we subjected 24-h-old pupae (referred to as 0-h of X-ray irradiation in this study, see “Methods” section) to various doses of X-ray irradiation. We use fecundity (number of eggs laid per female) and fertility (egg hatch rate) as two indicators for sterility. As expected, the fecundity of irradiated females exhibited a dose-dependent response (Fig. 1A). Between doses of 10 to 20 Gy, the average number of eggs laid per female significantly decreased from 83.5 eggs/female at 10 Gy to 0.89 eggs/female at 20 Gy, compared to 101.2 eggs/female in those non-irradiated (0 Gy) controls. At doses of 25 Gy or higher, essentially none of the irradiated females produced eggs, except for one female that produced 4 eggs (Fig. 1A), and none of these eggs hatched (Fig. 1A, B). Therefore, a dose of 25 Gy on 24-h-old pupae is sufficient to induce complete sterility in females under laboratory conditions.

We also investigated these X-ray doses on male fertility by crossing irradiated males with wild-type females. Expectedly, no difference in the fecundity of these wild-type females was observed across different experimental groups (Fig. 1C). However, the egg hatch rate exhibited a clear dose-dependent reduction (Fig. 1D), dropping from 28.3 at 10 Gy to 0.98% at 35 Gy, in line with previous reports [35–37]. In the subsequent experiments, we chose the X-ray dose of 30 Gy to ensure complete female sterilization after irradiation.

### Irradiated mosquitos show no cost to longevity under stress conditions

**Fig. 2:**
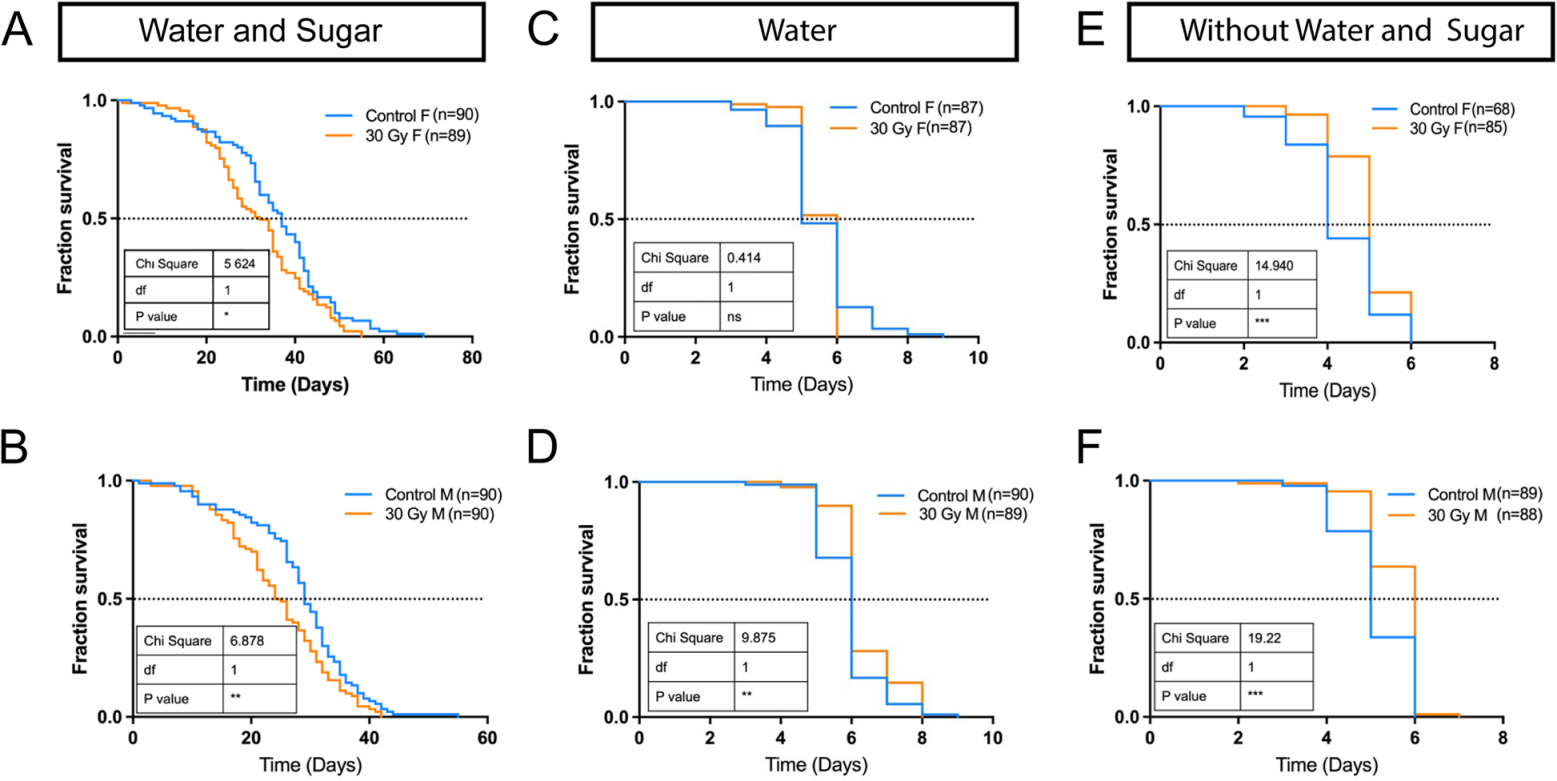
Effects of 30 Gy X-ray irradiation on *Aedes* longevity. Kaplan-Meier survival curves of female (**A**) and male (**B**) adults derived from irradiated pupae with constant water and sugar supply. Kaplan-Meier survival curves of female (**C**) and male (**D**) adults derived from irradiated pupae with water supply only. Kaplan-Meier survival curves of female (**E**) and male (**F**) adults derived from irradiated pupae without water and sugar supply. The number of samples in each figure is shown in the graph key. Pairwise comparisons of survival curves are performed by log-rank (Mantel-Cox) test; *** (*p* < 0.001), ** (*p* < 0.01), * (*p* < 0.05), ns (not significant)

To evaluate the effect on mosquitoes’ longevity, we examined the lifespan of mosquitoes that emerged from pupae exposed to 30 Gy irradiation. Under optimal laboratory conditions where constant water and sugar were provided, both irradiated females and males showed a significant decrease in lifespan, compared to non-irradiated (0 Gy) controls (Fig. 2A, B). Previous publications estimated that the average lifespan of *w*AlbB-SG males under the field condition was around 4.5 days [26, 38], significantly shorter than those under laboratory conditions (about 24.5 days). We next investigated the lifespan of those irradiated mosquitoes under stress conditions resembling field conditions by removing sugar or water or both. Under the water-only condition (without sugar supply), the lifespan of irradiated females was comparable to those of control females (Fig. 2C). Under this condition, irradiated males, however, survived better than control males (Fig. 2D). Of note, under the extreme condition of depletion of both sugar and water, there was an increase in lifespan observed in both irradiated males and females, compared to the controls (Fig. 2E, F). These results thus showed that a dose of 30 Gy irradiation does not cause a reduction to mosquitoes’ lifespan under stress conditions.

### X-ray irradiation disrupts ovariole maturation in female Ae. aegypti

Although irradiation also induces sterility in females [35], the underlying mechanism is not fully understood. Previous publications reported altered gross ovary morphology in irradiated *Aedes* females, but did not provide a detailed analysis [29, 37]. Therefore, we investigated the underlying cause of sterility in irradiated female mosquitoes.

**Fig. 3:**
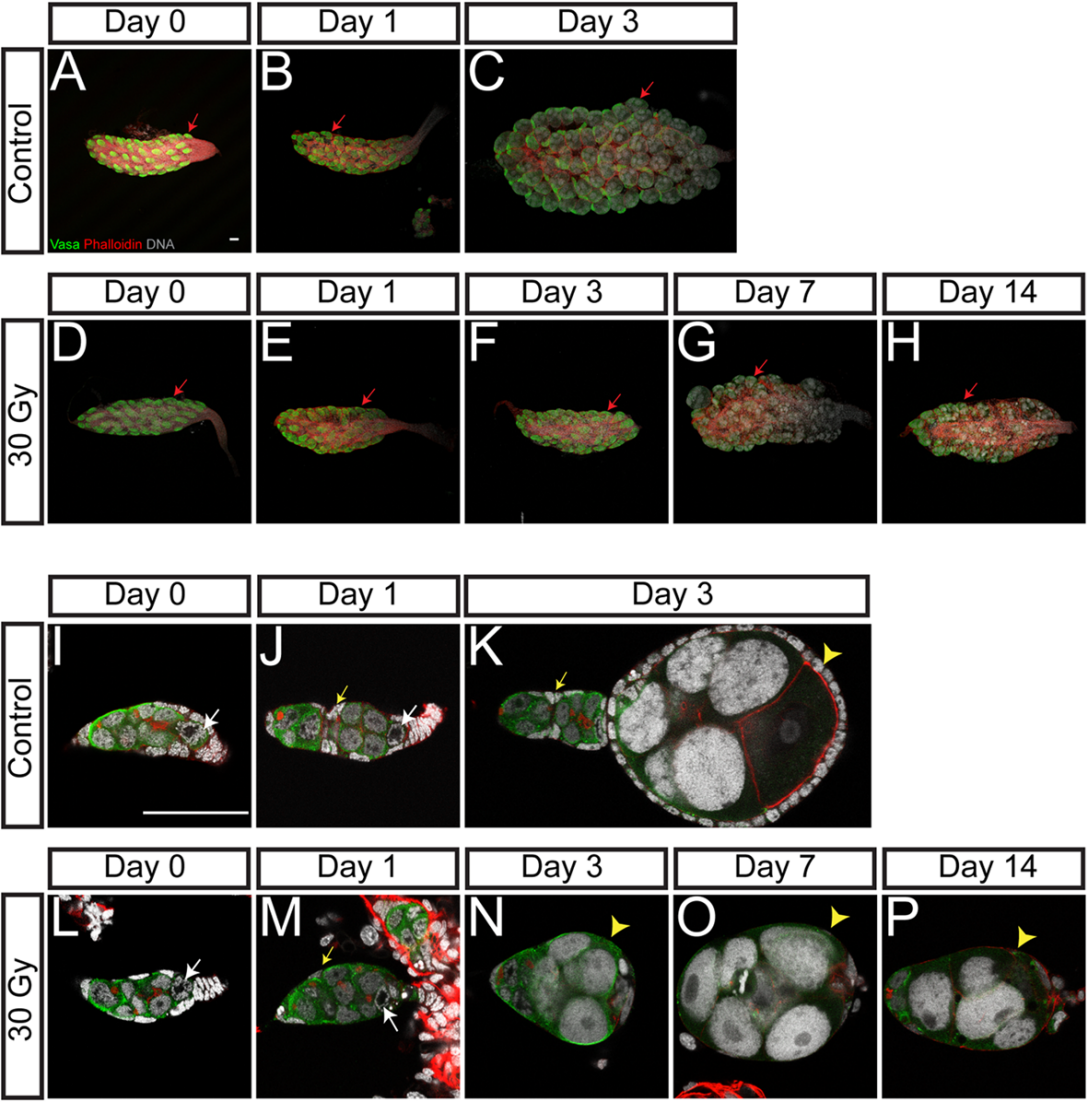
X-ray irradiation disrupts the development of ovary. Representative confocal microscopy images of ovaries (**A–H**) and ovarioles (**I–P**) with Vasa (Green), Phalloidin (Red), and DNA (Grey) staining at day 0 (**A** and **I**), day 1 (**B** and **J**), and day 3 (**C** and **K**) in control females, and day 0 (**D** and **L**), day 1 (**E** and **M**), day 3 (**F** and **N**), day 7 (**G** and **O**), and day 14 (**H** and **P**) in irradiated females after irradiation. **A–H** Red arrows indicate one ovariole (green marked by Vasa staining) in each sample. **I,J,L** and **M** White arrows indicate the future oocyte as indicated by the condensed chromosome. Yellow arrows in **J** and **K** indicate follicular cells bisecting the germarium into two segments, and the yellow arrow in **M** shows that no follicular cells bisect the germarial region of the ovariole from irradiated females. **K**, **N-P** follicular cells are present in control follicles (yellow arrowhead in **K**) but absent in ovarioles from irradiated females (yellow arrowheands in **N–P**). Scale bars in **A** (for **A–H**) and **I** (for **I–P**) are 50 µm

The formation of *Aedes* ovariole, similar to that of *Drosophila melanogaster*, begins in the late larval stage [39]. In control pupae, the pre-ovarioles (defined as the immature form of ovariole) formed and resided on the surface of the ovary (Fig. 3A for ovary and 3I for ovariole). The ovary grew in size after female emergence and matured by day 3 (Fig. 3B, C). A closer examination revealed that in 24-h-old pupae (0-h of irradiation), the pre-ovarioles in *Aedes* mosquitoes were elongated in shape, with several germline cells situated within the tubular structure formed by surrounding somatic cells (Fig. 3I). The future oocyte of the first germline cyst was evident by its condensed chromosome and located at the posterior end of the germ cell cluster (Fig. 3I, white arrow). During maturation, the germ cells proliferated, and the somatic cells divided and bisected the ovariole to form two segments, an anterior portion filled with undifferentiated germ cells (covered by a layer of inner germarial sheath cells, IGS cells) and the posterior part with a developing germline cyst covered by a single layer of follicular cells, which eventually budded out of the germarium to become the primary follicle (Fig. 3J). After female emergence, the primary follicle continued to grow, expanded its volume, and reached the mature (previtellogenic arresting) stage by day 3, while the secondary follicle formed in the germarium (Fig. 3K).

To understand the effects of X-ray irradiation, we traced the development of the ovary and ovariole from day 0 to day 14 post-irradiation. As expected, in pupae right after irradiation (day 0, about one hour after the irradiation), the ovaries of irradiated females were morphologically similar to those of control ovaries (Fig. 3D, compared to 3A). By day 1 (24 h post-irradiation), no obvious overall morphological difference was observed in comparison to the control (Fig. 3E, compared to 3B). However, while control ovaries grew and matured by day 3, the ovaries of irradiated females did not grow and were similar in size to those of day 1 ovaries (Fig. 3F, compared to 3C). These ovaries remained underdeveloped even by day 14 (Fig. 3G, H, compared to 3C). A close-up examination, however, showed that ovariole segmentation of irradiated females was blocked at day 1 (Fig. 3M) compared to the ovarioles of non-irradiated controls (Fig. 3J). While control ovarioles contained two segments (Fig. 3J), no segmentation was observed for ovarioles of irradiated females (Fig. 3M). Of note, ovarioles of irradiated females contained fewer visible somatic cells than those of the controls, and in general, lacked the layer of follicular cells covering developing germ cell cyst (Fig. 3M, compared to 3 J). Interestingly, the development of the germline cyst in the ovarioles of irradiated females was not affected, as the oocyte of the first germline cyst was specified and located at the posterior position (Fig. 3M, white arrow), similar to that of the control ovarioles (Fig. 3J). Although the germline cyst was able to grow, as evidenced by the enlarged nurse cell nuclei, it failed to bud out of and separated from the germarium and eventually fused with the anterior germarium (Fig. 3N). Furthermore, while the germ cells in the primary follicle of the control ovarioles were surrounded by a single layer of follicular cells (Fig. 3K), in irradiated female mosquitoes, the germ cells of the primary follicle were not covered by a layer of follicular cells and were arrested in development (Fig. 3N–P). Meanwhile, the anterior part of the germarium shrank and did not produce the secondary follicle. A reduction of IGS cells was observed in the anterior segment of the ovarioles from irradiated females (Fig. 3N, compared to 3 K). In summary, these results show that the ovariole maturation was blocked by irradiation, likely due to the lack of somatic cells (including IGS and follicular cells), which consequently disrupts oogenesis, resulting in sterility.

### X-ray irradiation blocks proliferation and causes apoptosis during ovariole maturation

**Fig. 4:**
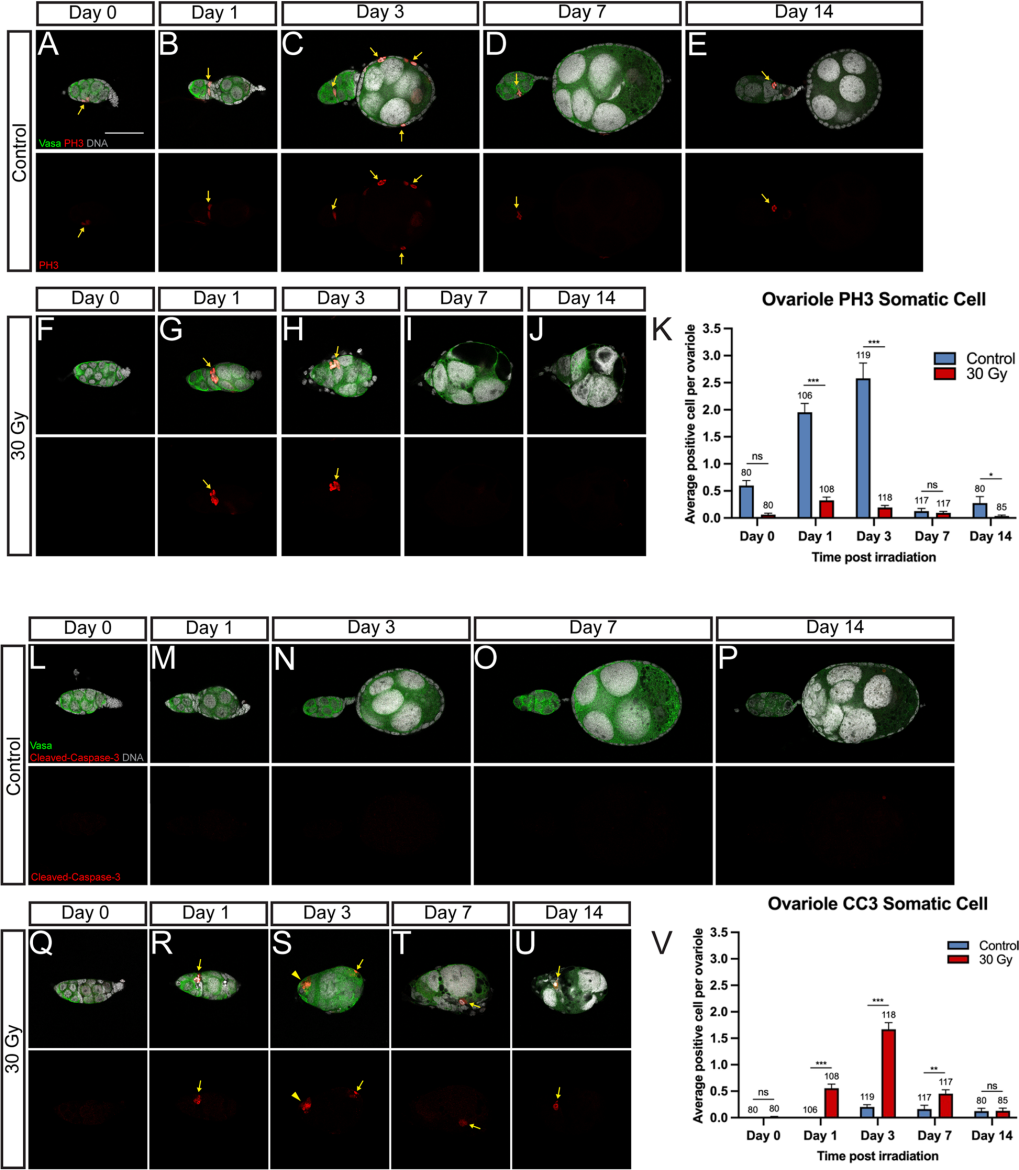
X-ray irradiation blocks ovariole maturation. **A–J** Representative confocal microscopy images of ovarioles with Vasa (Green), PH3 (Red), and DNA (Grey) staining at day 0 (**A**), day 1 (**B**), day 3 (**C**), day 7 (**D**), and day 14 (**E**) in controls, and at day 0 (**F**), day 1 (**G**), day 3 (**H**), day 7 (**I**), and day 14 (**J**) in irradiated females after irradiation. Yellow arrows indicate PH3-positive cells. **K** Quantification of the average PH3-positive **K**. **L–U** Representative confocal microscopy images of ovarioles with Vasa (Green), cleaved-Caspase3 (Red), and DNA (Grey) staining at day 0 (**L**), day 1 (**M**), day 3 (**N**), day 7 (**O**), and day 14 (**P**) in controls, and at day 0 (**Q**), day 1 (**R**), day 3 (**S**), day 7 (**T**), and day 14 (**U**) in irradiated females after irradiation. Arrows point to somatic cells. The arrowhead points to the germ cell. The scale bar in **A** (for all image panels) is 50 µm. **V** Quantification of the cleave-Caspase3-positive somatic cells per ovariole. Error bars in **K** and **V** represent mean ± SEM. Comparisons of control and irradiated samples are performed by *t*-test; *** (*P* < 0.001), ** (*P* < 0.01), * (*P* < 0.05), ns (not significant). The number of samples in each group is shown above the *X*-axis

The lack of somatic cells (IGS and follicular cells) in the ovarioles of irradiated females could be caused by a defective proliferation and/or apoptosis induced by irradiation. To understand the cause of these irradiation-induced ovariole maturation defects, we used anti-Phospho-Histone H3 (pSer10), PH3, and anti-cleaved Caspase3, CC3, antibodies to detect the proliferative or apoptotic cells, respectively, in these ovaries. In the control ovaries, active proliferation was observed in the somatic cells residing in the middle of the germarium during the process of germarium segmentation (Fig. 4A, B, and K), indicating that both cell proliferation and migration are required to bisect the germarium into the anterior section with undifferentiated germ cells and the posterior compartment containing a developing germline cyst (the primary follicle). Subsequently, during the growth stage of the primary follicle, extensive proliferation of the follicular cells was observed (Fig. 4C, K). After reaching the mature (previtellogenic arresting) stage, no active proliferation was detected in the primary follicle, and only limited proliferation of the follicular cells was observed in the germarium (Fig. 4D, E, and K). On the contrary, a significant reduction of proliferating follicular cells was detected in the irradiated samples. The reduced proliferation was observed as early as day 0 (several hours after irradiation), although the difference is not statistically significant (Fig. 4F, K). However, a significant reduction of proliferating follicular cells was detected during the ovariole maturation process (Fig. 4G–K).

In addition to the reduced proliferation, apoptosis resulting from irradiation might also contribute to the deformative ovarioles. In controls, no apoptotic cells, including germ cells and somatic cells (IGS and follicular cells), were observed at day 0 or day 1 ovarioles, and very few apoptotic follicular cells were detected in the ovarioles during the ovariole maturation process (Fig. 4L–P, and V). However, low levels of the apoptotic signal were occasionally detected as early as a few hours after irradiation, but a significant increase in apoptotic cells was observed in day 1, day 3, and day 7 ovarioles of irradiated females (Fig. 4R–V). On day 1, apoptotic cells were somatic cells and mainly detected in the middle germarial region where active proliferation was previously observed during germarium segmentation (Fig. 4B, R), in line with the notion that the mitotic cells are more vulnerable to irradiation [40]. On day 3, ovarioles contained few somatic cells, and apoptosis was frequently detected in these remaining follicle cells (Fig. 4S). It is difficult to distinguish the somatic cell type (IGS or follicular cells) in these ovarioles due to the deformed morphology and lack of cell type markers (Fig. 4S). Of note, apoptosis was also detected in germ cells at this time point (Fig. 4S, yellow arrowhead), presumably as the consequence of a lack of support from these somatic cells and consistent with the observed shrinkage of the germarium. From day 7 onwards, the number of apoptotic cells reduced (likely due to the significant reduction of somatic cells by this time point), and most of them were follicular cells (Fig. 4T–V). Collectively, X-ray irradiation caused a reduction of somatic cell proliferation and an elevation of apoptosis in both somatic cells and germ cells, which led to the blockage of ovariole maturation and consequently resulted in the sterility of irradiated females.

### X-ray irradiation does not interrupt the development of Aedes aegypti testes

**Fig. 5:**
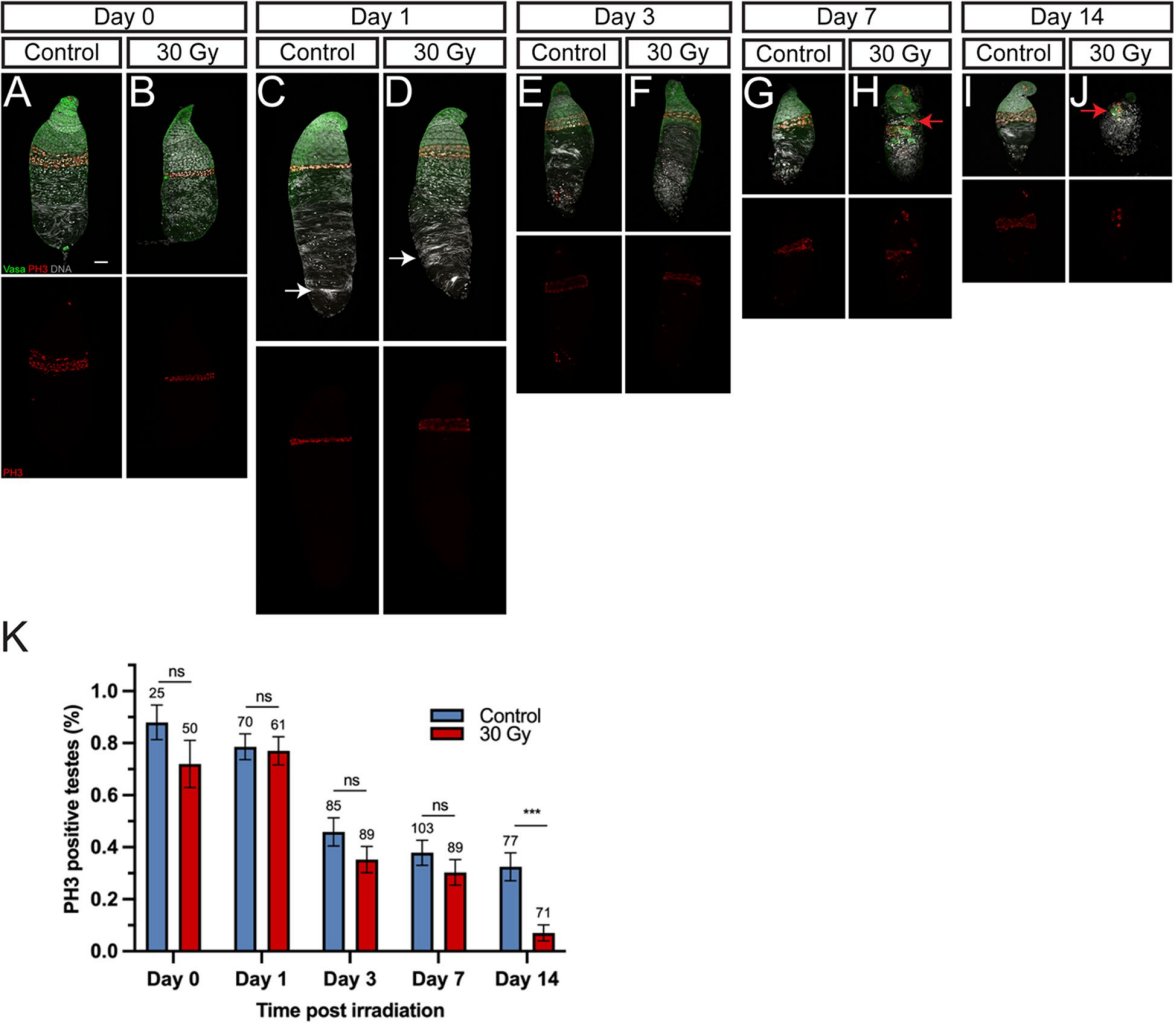
X-ray irradiation does not interrupt the development of testis. **A–J** Representative confocal microscopy images of testes with Vasa (green), PH3 (red), and DNA (grey) staining at day 0 (**A**), day 1 (**B**), day 3 (**C**), day 7 (**D**), and day 14 (**E**) in controls, and at day 0 (**F**), day 1 (**G**), day 3 (**H**), day 7 (**I**), and day 14 (**J**) in irradiated males after irradiation. White arrows in **C** and **D** indicate mature sperm. Red arrows in **H** and **J** indicate Vasa-positive germ cells located at ectopic position (compared to **G** and **I**). The scale bar in **A** (for all image panels) is 50 µm. **K** Percentage of PH3-positive testes. Comparisons of control and irradiated samples are performed by Fisher’s exact test; **** (*P* < 0.0001), ns (not significant). The number of samples in each group is shown above each column

Given the strong reduction of fertility in irradiated males at 30 Gy, we intended to understand the reason behind this sterility. *Ae. aegypti* testis development, similar to that of *D. melanogaster*, occurs earlier than ovary development ([41] and data not shown). By 24-h-old pupal stage (or day 0 of irradiation), the testes already contain germ cells at different differentiating stages (Fig. 5A), arranged in a gradient from the anterior tip (germline stem cells) to the posterior end (elongating spermatids). Testes continued to grow and by day 1, mature sperms were present at the posterior end (white arrow in Fig. 5C). From day 3 to day 14, testes progressively reduced their size (Fig. 5E, G, and I), presumably due to the usage of sperms during the mating process. Surprisingly, no obvious morphological change was observed in the day 0 to day 3 testes of irradiated males, compared to those of the control testes (Fig. 5A–F). Only by day 7 and day 14, some testes of irradiated males exhibited some abnormal patches of Vasa-positive germ cell clusters at ectopic positions in the middle of the testes (Fig. 5G–J, red arrows, and Additional file 1: Fig. S1A-J). A reduction of Vasa-positive early-stage germ cells was also observed in day 14 testes of irradiated males. Hence, unlike its strong effect on the ovariole morphology, X-ray irradiation did not have a strong impact on the overall structure of the testes during its mating competitive stage. It is worth noting that testes from irradiated males exhibited a reduction in germ cell proliferation measured by PH3, compared to the controls, although it is not statistically significant between day 0 to day 7 (Fig. 5A–K), and only by day 14, testes of irradiated males showed significantly reduced germ cell proliferation (Fig. 5K). Furthermore, more testes from irradiated males contained some apoptotic cells when compared to control testes from day 3 onwards (Additional file 1: Fig. S1K). Collectively, X-ray irradiation on 24-h-old pupae has a limited effect on the overall morphology of testes during its early stage.

### X-ray irradiation causes chromosomal damage to sperm

**Fig. 6:**
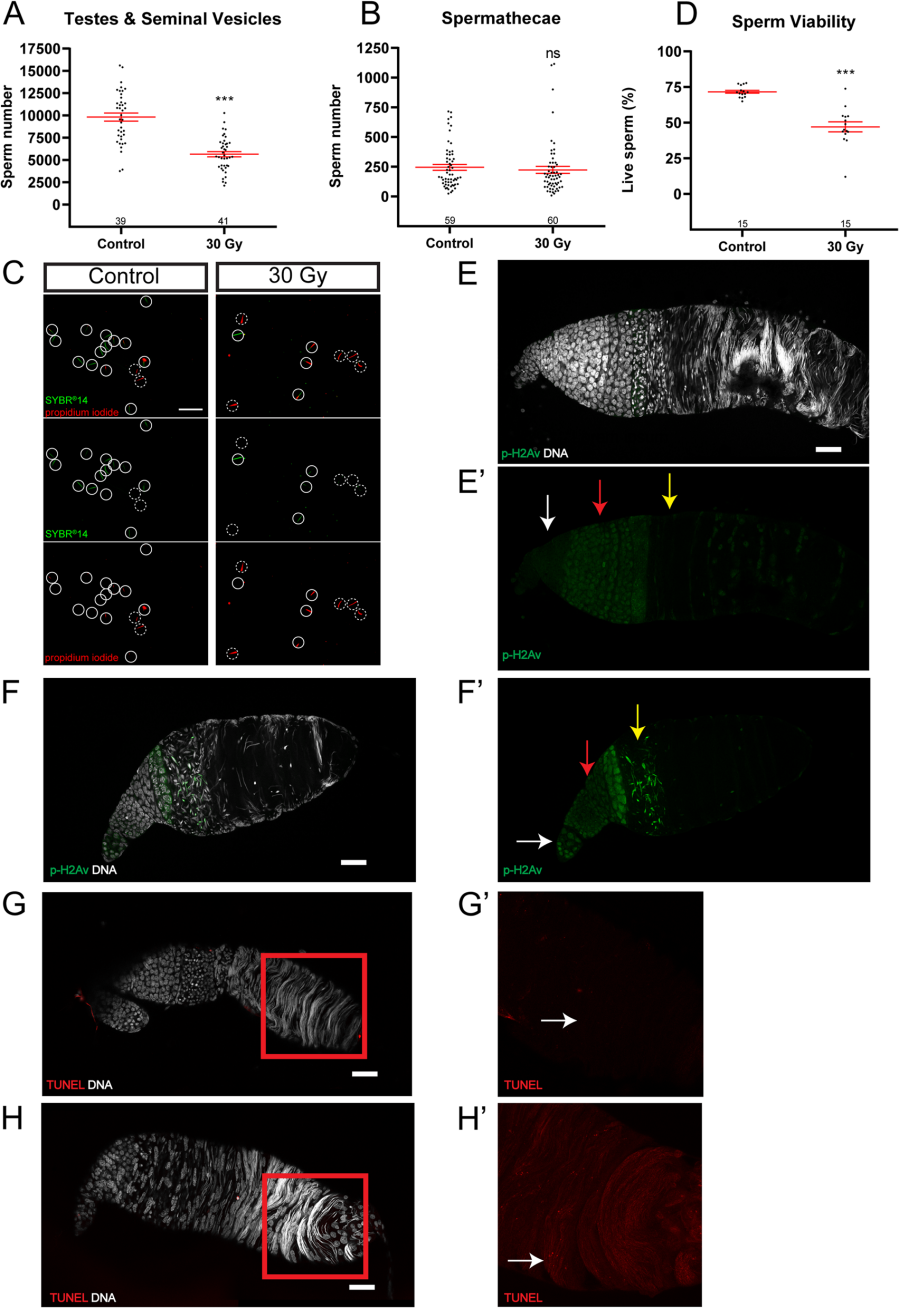
X-ray irradiation affects sperm viability. **A** Quantification of sperm number in testes and seminal vesicle of wild-type and 30 Gy irradiated males. **B** Quantification of sperm number in spermathecae of wild-type females mated with wild-type or 30 Gy irradiated males. **C** Representative microscopy images of live sperm (Green) or dead sperm (Red) of wild-type and 30 Gy irradiated males. **D** Percentage of live sperm of wild-type or 30 Gy irradiated males. **E-F** Anti-H2Av staining only labels meiotic spermatocytes (red arrow) in wild-type testis. H2Av signals are also detected in GSCs, spermatogonia (white arrow), and differentiating spermatids (yellow arrow) in irradiated males (**F**). **G–H** TUNEL staining does not detect consistent signals in wild-type testes (**G**), but is elevated (decorating spermatid DNA, white arrow) in irradiated males (**H**). **G’** and **H’** are close-up views for the regions indicated in **G** and **H**. Comparisons of controls and irradiated samples are performed by paired *t*-test; *** (*P* < 0.001), ns (not significant). Error bars represent mean ± SEM. The number of samples in each group is shown above the X-axis

Next, we investigated whether X-ray irradiation affected sperm quantity and quality, which could have led to the observed male sterility. We first checked whether irradiation might affect the production of sperm, although there was no overall abnormality in the testis morphology. While each control male contained about 10,000 sperm, the irradiated male on average harboured significantly fewer sperm in its testes and seminal vesicles (Fig. 6A). Males, in general, produce an excess number of sperm via continuous spermatogenesis. Thus, the 50% reduction in sperm in irradiated males might not explain the high sterility observed. To address this, we examined the number of inseminated sperm in spermathecae of females mated with control or irradiated males. Interestingly, there was no significant difference in terms of sperm quantity in spermathecae of females mated with control or irradiated males (Fig. 6B). These results suggest that irradiation indeed affects spermatogenesis (by reducing sperm production) but does not affect mating and insemination between wild-type females and irradiated males. It further indicates that reduced sperm production in irradiated males is not the underlying cause of the strong sterility observed. We moved on to examine the portion of dead/damaged sperm using a live/dead sperm viability assay. Notably, the sperm samples of irradiated males showed a higher portion of dead or damaged sperm than those of control males (Fig. 6C), and the sperm survival rate dropped from 71.7% in controls to 47.0% in irradiated samples (Fig. 6D), indicating that quality of sperm is strongly affected by irradiation. To further address DNA damage in these irradiated samples, we examined two additional double-strand break (DSB) markers, p-γ-H2Av and TUNEL staining. γ-H2Av is a conserved histone H2A variant of human γ-H2Ax and its phosphorylation serves as an indicator for DNA DSB. *Ae. aegypti* genome harbours one functional ortholog of the human H2A variant (AAEL012499) [42, 43]. As expected, in the control testes, p-γ-H2Av was only detected in meiotic spermatocytes but not in other germ cells (Fig. 6E). In the irradiated samples, ectopic p-γ-H2Av was detected in most germ cells, including GSCs, spermatogonia, and early differentiating spermatids (Fig. 6F). In the control testes, TUNEL staining did not detect consistent signals above the background (Fig. 6G), while its signal decorated many differentiating spermatids in the irradiated samples (Fig. 6H), showing DNA damage in these spermatids. These results together demonstrate that X-ray irradiation induces chromosomal damage to male germ cells. Hence, the observed male sterility after pupal irradiation is likely due to a decline in sperm quality.

### Wolbachia-infected Ae. aegypti strain is more sensitive to X-ray irradiation

**Fig. 7:**
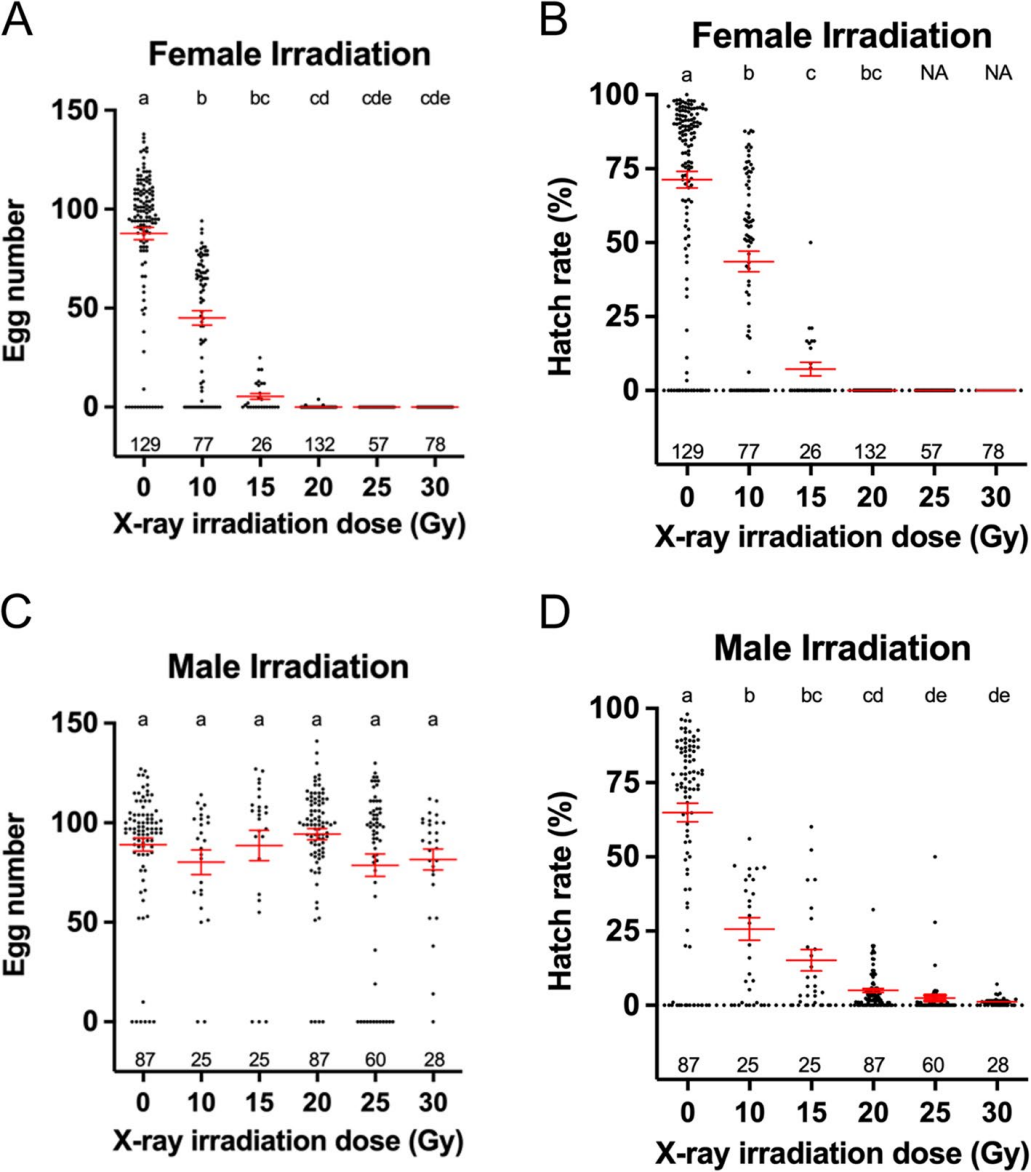
Effect of irradiation dose on the fecundity and hatch rate in *w*AlbB-SG. Effects of various doses of X-ray irradiation on egg production (**A**) and hatch rate (**B**) for irradiated *w*AlbB-SG females mated with wild-type *w*AlbB-SG males. Effects of various doses of X-ray irradiation on egg production (**C**) and hatch rate (**D**) for wild-type *w*AlbB-SG females mated with irradiated wAlbB-SG males. The number of samples in each group is shown above the X-axis. Error bars represent median and interquartile range. Kruskal-Wallis test and Dunn’s multiple comparisons post hoc test shows the difference between control and individual irradiation dose; doses not sharing the same letter are significantly different

So far, we have tested the working dose of X-ray irradiation during the pupal stage on wild-type *Ae. aegypti* (NEA-EHI strain) and investigated the cause of sterility in both female and male mosquitoes. Building upon these findings, we moved on to test the dose of X-ray irradiation on *w*AlbB-SG, the *Wolbachia*-infected *Ae. aegypti* (NEA-EHI strain) used in the IIT-SIT operational test under laboratory conditions. Our results showed that both egg number and hatch rate of irradiated *w*AlbB-SG females reduced significantly in a dose-dependent manner (Fig. 7A, B). *w*AlbB-SG females subjected to 25 or 30 Gy irradiation did not produce any eggs, only 4 out of 132 females subjected to a dose of 20 Gy irradiation laid few eggs and none of these eggs hatched (Fig. 7A, B). Thus, the minimal dose to induce complete sterility in females of *w*AlbB-SG strain is 20 to 25 Gy, lower than that of the wild-type strain (25–30 Gy), indicating that the *Wolbachia*-infected strain is likely more sensitive to X-ray irradiation than its parental *Ae. aegypti* (NEA-EHI strain). Similarly, it was reported that *Wolbachia*-infected *Ae. aegypti* lines (Brazil and Mexican strains) are more radiosensitive to irradiation than the uninfected ones [44]. As expected, *w*AlbB-SG females mated with irradiated *w*AlbB-SG males and produced a comparable number of eggs as *w*AlbB-SG females mated with control males (Fig. 7C). However, the hatch rate of these eggs showed a dose-dependent reduction, compared to the controls (Fig. 7D), which is consistent with the previous report [26]. Collectively, *w*AlbB-SG mosquitoes are likely more sensitive to X-ray irradiation, compared to the uninfected controls, and a lower dose of X-ray (20 to 25 Gy) is able to achieve complete female sterilization.

## Discussion

The use of SIT has been shown to be effective in suppressing vector populations, especially when implemented as part of an integrated vector management approach that incorporates other vector control tools [10, 12, 16]. The conventional SIT approach utilizes irradiation to render male mosquitoes sterile, and the success of this approach relies on the ability of sterile males to effectively compete with field males to mate with field females, resulting in these females laying non-viable eggs. On the other hand, the combined IIT-SIT utilizes CI to induce sterility in female mosquitoes, while irradiation is used to sterilize any *Wolbachia*-infected females that have slipped through the sex sorting process to prevent possible population replacement [24–27, 45]. Despite reduced fitness in irradiated male mosquitoes, several field trials have successfully demonstrated the efficacy of both approaches in suppressing the *Aedes* vector population across various field settings [24–27]. Determining the optimal dose range for the SIT approach depends on achieving the desired level of sterility while maintaining the competitiveness of irradiated males. Thus, a better understanding of the relationship between irradiation and mosquito biology is warranted.

In this study, we explore the relationship between X-ray irradiation dose and mosquito fecundity, fertility, and longevity to identify the minimum dose that could completely sterilize females with minimal effect on male performance traits. We chose to irradiate 24-h-old *Ae. aegypti* pupae for the following reasons: First, compared to adults, irradiation of the pupae stage is more feasible to handle under our laboratory conditions due to the complex issue associated with adult transportation from our insectary to the X-ray facility. Second, it has been documented that different developmental stages exhibit different sensitivities to irradiation, and irradiation at a later stage generally minimizes developmental defects and lessens the performance trait costs associated with the irradiation process. Similar to previous publications showing that irradiation at a late pupae stage could reduce mortality [36, 46, 47], our preliminary experiments revealed that exposing (L1–L4) larvae and early (0–3 h) pupae to 30 Gy irradiation resulted in high mortality rates (data not shown), whereas irradiation of same dose on late-stage pupae (24 h) showed very low mortality. Third, our previous study on *Ae. aegypti* germline development revealed that the late pupal stage represents the crucial phase for the maturation of ovaries [39]. These findings establish an optimal window for using irradiation to target the female germline for sterilization. In the SIT approach, higher irradiation doses, ranging from 35 to 70 Gy, are applied in order to achieve a 99% male sterility [37, 48–50]. It is worthwhile to note that most of these studies use a Gamma radiation source. Compared to this, our results show that a lower dose of X-ray irradiation on 24-h-old pupae is sufficient to induce complete female sterility. Under our laboratory conditions, an X-ray irradiation dose of 25 Gy was effective in inducing complete sterility in female mosquitoes. Although this dose is lower than the 30 Gy dose used in other studies [24, 26], it still has a strong impact on male fertility [35, 37]. However, under stress conditions, where only water was provided to the mosquitoes, the irradiated mosquitoes exhibited a minor survival advantage, marked by a slight increase in longevity. Although the detailed mechanisms warrant further investigation, these unexpected results could potentially stem from the activation of the beneficial pathways triggered by irradiation, such as the antioxidant pathways, and the DNA repair mechanisms [51–55]. We would also like to point out that male mating competitiveness, another important indicator of performance traits, has not been addressed in our current study.

Ionizing radiation causes molecular bonds to break and generates free radicals within biological tissues, significantly affecting the chromosomal integrity of dividing cells [52]. Generally, mitotic cells are more vulnerable to ionizing radiation. In the SIT method, irradiation-mediated male sterility is attributed to the damage to both proliferating germ cells and somatic cells. Irradiation-induced dominant lethal mutations in germ cells, which do not affect sperm maturation but have a sterilization effect on fertilized embryos, are proposed to be the main cause of male sterilization in the SIT approach [12]. In contrast, the underlying cause of female sterility induced by irradiation remains largely unknown in mosquitoes. Two previous studies showed that the ovaries of irradiated *Aedes mosquitoes* are shorter than those of non-irradiated controls [29, 37]. Similarly, our results show that the overall morphology of irradiated *Ae. aegypti* ovaries were smaller and deformed with age compared to those of non-irradiated controls. While the control ovaries grew after female emergence and matured by day 3, the ovaries of irradiated females showed delayed growth for an extended period and eventually degenerated. A thorough analysis of proliferative and apoptotic cells of ovarioles provides some insights into the observed phenotype. In wild-type mosquitoes, ovariole growth and maturation are mainly facilitated by the cellular growth (through endoreplication without mitotic divisions) of nurse cells in the primary follicle along with the concurrent proliferation of follicular cells in both germarium and the primary follicle. In the ovaries of irradiated females, nurse cells of the primary follicles could also undergo endoreplication to increase their nuclear size, but the proliferation of follicular cells was strongly inhibited. Concurrently, apoptosis occurred in the follicular cells within both the germarium and the primary follicle. The combined effects of halted proliferation and induced apoptosis in the follicular cells lead to the gradual loss of somatic cells, impediment of ovariole maturation, and ultimately result in deformed ovarioles. At a later time point, germ cell death was also observed, presumably due to the absence of the follicular cells. Therefore, the sterility of females induced by pupal irradiation is initiated and caused by the disruption of the follicular cells during the maturation and growth of the ovariole.

In irradiated males, we did not observe any obvious structural or morphological changes in the testis, particularly during the early mating competitive stages. Based on these observations, it seems that the sterility induced by pupal irradiation in males is a mechanistically distinct process from that in females. Indeed, further analyses reveal that, although irradiated males produced only about 50% of mature sperms compared to non-irradiated males, the number of inseminated sperms in female spermathecae showed no difference, suggesting that a reduction in sperm production is not the cause of observed sterility. However, according to the live/dead sperm viability assay, a significant increase in damaged and dead sperm was observed in the irradiated samples. In line with the notion of chromosomal damage induced by irradiation, staining of anti-cleaved Caspase 3 was detected in the differentiating zone which contains spermatocytes, spermatids, and spermatozoa. Since females are generally inseminated by live sperms, it is likely these inseminated sperms harbour some chromosomal damages induced by irradiation, which leads to lethal embryos after fertilization, similar to previous reports in the SIT approach [40]. In line with this, p-γ-H2Av and TUNEL staining, two DSB markers, were ectopically detected in male germ cells of irradiated males. Taken together, these findings support the hypothesis that dominant lethal mutations in sperms, resulting from irradiation, are the primary cause of the observed male sterility, which in turn leads to embryonic mortality after fertilization [56–58]. Therefore, the underlying mechanisms of irradiation-induced sterility differ between males and females.

An important issue of the IIT-SIT approach is the performance trait cost of males incurred by irradiation. Indeed, some performance trait cost on male mosquitoes has been observed in these operational tests [24, 26, 29, 59, 60]. Based on our results on the field strain of *Ae. aegypti* (NEA-EHI strain), the parental strain of *w*AlbB-SG, we conducted experiments to search for the minimum X-ray dose with full female sterilization effect but with low performance trait cost to *w*AlbB-SG male mosquitoes. Our results show that *w*AlbB-SG strain is likely more radiosensitive compared to its parental NEA-EHI strain. The minimum dose to induce full sterility in *w*AlbB-SG females is 20–25 Gy, lower than the dose of 25–30 Gy identified for the NEA-EHI strain. Similar results have been previously reported for *Ae. aegypti* (Brazil and Mexican strains).

It is worth noting that the settings and procedures of X-ray irradiation and pupae handling in the operational field trials are far more complex and significantly different compared to the laboratory conditions used in this study. For instance, 24-h-old pupae used in this study were collected within a 6-h window after pupation, it is not feasible to collect pupae within such a short period in a large-scale operation. Furthermore, pupae irradiation was conducted in a small petri dish, so uniformed X-ray irradiation is achievable in this study; it is, however, difficult when handling a massive number of pupae in a large-scale setting. In the future, we will further address the experimental parameters for large-scale pupae irradiation to minimize the performance trait cost to *w*AlbB-SG males in the IIT-SIT programme.

## Conclusions

X-ray irradiation has been used in various mosquito control methods, including SIT and IIT-SIT, to sterilize mosquitoes (males in the SIT and females in the IIT-SIT). However, the underlying mechanisms for these irradiation-induced male and female sterilities remain unclear. Our results, presented in this study, reveal that X-ray irradiation at 24-h-old pupae induces male and female sterility via different mechanisms. In males, it likely acts directly on germ cells via chromosomal damage (dominant lethal mutations), while in females, it targets somatic supporting cells (IGS and follicular cells), resulting in disrupted ovarian maturation and consequently leading to female sterility. Our findings may help in the design of irradiation-based vector control methods.

### Limitations of this study

In this study, we have tested and identified the minimum dose of X-ray irradiation for complete female sterilization, with some effects on male mosquitoes, in *Ae. aegypti* (NEA-EHI strain). Since several factors, including different genetic backgrounds of mosquitoes, radiation source, irradiation rate, oxygen levels, temperature, and pupae age, could significantly influence the outcomes of irradiation, caution should be exercised when extrapolating our results to other *Ae. aegypti* strains.

Similar to a previously published study, our data indicate that under laboratory conditions, *Wolbachia*-infected mosquitoes (*w*AlbB-SG in this work) are more radiosensitive than their uninfected counterparts. It is important to note that these two lines (*w*AlbB-SG and its parental NEA-EHI strains) are not isogenic lines and likely have some genetic variations in their backgrounds, which could potentially contribute to the observed differences in radiosensitivity. Additional experiments are necessary to further investigate this conclusion.

## Methods

### Mosquito breeding

The laboratory colonies of *Ae. aegypti* (NEA-EHI strain) and *Wolbachia*-infected *Ae. aegypti* (*w*AlbB-SG) [26] used in this study originated from the National Environment Agency (NEA), Singapore. Colonies were reared in an environmental chamber at a constant temperature of 28 °C ± 1 °C, relative humidity of 80% ± 5%, and a photoperiodic regime of 12:12 h (light:dark). Mosquito eggs, aged between 2 and 4 weeks post-egg laying, were hatched in sterile water by vacuum for 15 min. Newly hatched L1 larvae were reared at a density of 2.5 mL/larvae (200 larvae in 500 mL) in sterile water. The larvae were fed with a daily diet consisting of a mixture of fish food (TetraMin Tropical Flakes Fish Food) and brewer’s yeast (yeast instant dry blue/bruggeman) in a 2:1 ratio. The feeding regimen was as follows: 25 mg (day 1), 32 mg (day 2), 56 mg (day 3), 130 mg (day 4), 200 mg (day 5), and 100 mg (day 6). Half of the daily food ration was given in the morning, and the other half in the evening.

### Mosquito pupae X-ray irradiation

Mosquito pupae aged between 0 and 6 h were collected, with the 3-h time point considered as 0 h of the experiment (making the pupae 0 ± 3 h old). These pupae were further aged for 24 h. Subsequently, 200 pupae (100 male pupae and 100 female pupae) were transferred into a 100 × 20 mm petri dish with 20 mL sterile water. The dish was sealed with parafilm, labelled, and transported to the EHI facility for irradiation. Prior to irradiation, these 200 pupae were transferred into a new petri dish (94 × 16 mm, Cat: 633,181, Greiner Bio-One) containing 20 mL sterile water to create a single layer of pupae in the water, preventing overcrowding during irradiation (refer to Additional file: Fig. S2). The Radsource RS2400V X-ray irradiator was used to irradiate all mosquito pupae in the study (Rad Source Technologies Inc, USA). Radiation dosimetry details are provided in the next section. Pupae were irradiated at 10, 15, 20, 25, 30, or 35 Gy (experiment specific), after which the pupae were transported back to the insectary located at Temasek Life Sciences Laboratory and were visually sorted by sex. Male and female pupae were separately placed into Bugdorm rearing cages, each supplied with one vial of 10% sucrose and one vial of sterile water. Additionally, for each experiment, an extra petri dish of 200 pupae (100 males and 100 females) packed under the same condition was transported to the EHI facility but did not undergo X-ray radiation, serving as the control (0 Gy). The breeding and handling (sex separation, transportation, irradiation, post-irradiation experiments) of *Ae. aegypti* (NEA-EHI strain) and *Wolbachia*-infected *Ae. aegypti* (*w*AlbB-SG) were conducted in parallel.

### X-ray irradiator and dosimetry system

The Radsource RS2400V X-ray irradiator, equipped with X-ray tube model of Quastar DT-1084, was used to irradiate all mosquito pupae in the study (Rad Source Technologies Inc, USA). The system comprises six individual X-ray containers that rotate around the central X-ray tube (Additional file: Fig. S3) to ensure uniform X-ray exposure. An ionization chamber (10X6-0.18, RadCal Corporation, USA) and a digitizer module (ADDM-plus Accu-Dose, RadCal Corporation, USA) were used as a reference dosimetry system to measure the dose rate. During the dose rate measurement, the ionization chamber was fixed at the centre of the X-ray canister by a customized acrylic holder to simulate X-ray irradiation exposure by mosquito pupae at a similar position (Additional file: Fig. S4). The irradiation doses used in this study were set by adjusting the exposure time based on the dose rate according to the manufacturer. To simulate the pupae irradiation setup during the dose mapping, three Petri dishes (94 × 16 mm, Cat: 633,181, Greiner Bio-One) were stacked together, and 20 mL sterile water was added to the middle Petri dish. A piece of Gafchromic™ film (Ashland Advanced Materials, USA) was inserted in between the middle and bottom petri dish and the entire setup (Additional file: Fig. S5) was placed into one of six X-ray canisters for irradiation at a predetermined dose. The dose map and dose uniformity (DUR) of the X-ray irradiator was determined by scanning the Gafchromic™ film using a flatbed scanner (Canon LiDE 400) post-irradiation. The analysis involved assessing the colour channel information from the scanned image within the effective pupae irradiating area (Additional file: Fig. S6). The scanning and analysis method adhered to the procedure recommended by FAO/IAEA (available ta https://www.iaea.org/sites/default/files/dose-mapping-gafchromic-2020-11-02.pdf).

### Mosquito fecundity and fertility test

Adult mosquitoes were provided with one vial of 10% sucrose solution and one vial of sterile water, both of which were replaced twice a week. To evaluate the impact of irradiation on females, thirty irradiated virgin females were mated with thirty non-irradiated, non-transported wild-type virgin males for 3 days in a 17.5 × 17.5 × 17.5 cm cage (BugDorm-4S1515 Insect Rearing Cage). After mating, female mosquitoes were fed rabbit blood using a Hemotek 5W1 membrane feeding system (Hemotek.co, UK). Non-blooded females were subsequently removed. Two days after the blood meal, females were aspirated out of the cages, briefly knocked down on ice, and individually transferred into a large Drosophila vial (28.5 × 95 mm, VWRI734-1255) containing one piece of filter paper as the oviposition substrate and 3 mL of sterile water. The vial was then sealed with a cotton plug, and female mosquitoes were allowed to oviposit for 3 days. The females were then removed and the eggs were counted. Eggs were hatched 2 days later by adding 20 mL of hatching solution (0.37 g Luria–Bertani LB broth powder and 0.07 g Bruggeman Instant Yeast granule in 1 L water). The hatched larvae were counted on the following day. The fecundity was measured by the number of eggs counted and the hatch rate was calculated by the number of larvae divided by the number of eggs. To assess the impact of irradiation on males, each cage contained thirty irradiated males and thirty non-transported wild-type virgin females. The control (0 Gy) cage for irradiated females consisted of thirty transported non-irradiated virgin females crossed with thirty non-transported wild-type virgin males and the control (0 Gy) group for irradiated males consisted of thirty transported non-irradiated virgin males crossed with thirty non-transported wild-type virgin females. Two sets of experiments were conducted for each test. The number of eggs and hatched larvae were counted twice by two different individuals to minimize human errors.

### Mosquito adult survival test

To determine the impact of irradiation on mosquito longevity, thirty 1-day-old irradiated males or females from non-irradiated control or each irradiation dose were transferred to 17.5 × 17.5 × 17.5 cm cages (BugDorm-4S1515 Insect Rearing Cage) separately and provided with 10% sucrose solution and sterile water. To simulate field conditions with limited/no food (sugar) access, two stress conditions were tested: one, mosquitoes without access to both sucrose and sterile water and two, mosquitoes with access to sterile water only. Mortality was monitored and recorded daily until all mosquitoes had died. Two independent tests were conducted for each condition with the exception of the water-only stress condition which only tested one batch of mosquitoes.

### Tissue dissection and immunostaining

To study the morphology of the germline and its development post-irradiation, male or female pupae/mosquitoes were dissected at the timepoints of 8 h/16 h/24 h/36 h/48 h/3 day/5 day/7 day/10 day/14 days after irradiation. The dissection, fixing, and immunostaining protocols followed our previously published protocols for Drosophila tissues [61]. In brief, the testes or ovaries were dissected in ice-cold PBS and fixed with 4% formaldehyde (diluted in PBS) for 20 min. The tissues were rinsed and washed with PBT (PBS with 0.1% Triton X-100) for 30 min before incubating with 5% NGS (new-born goat serum diluted in PBT) blocking solution for 30 min, after which the tissues were incubated with the primary antibody in 5% NGS overnight at 4 degree. On the following day, the tissues were rinsed and washed with PBT for more than 30 min before incubating with the secondary antibody conjugated with Alexa Fluor 555, 488, or 633 (diluted in PBT, Jackson ImmunoResearch, 1:200) for 2–4 h at room temperature. The tissues were subsequently washed with PBT for 30 min and incubated with DNA staining dye (diluted in PBT) for 1 h. The solution was then removed, and the tissues were stored in the VectaShield mounting medium (Vector Laboratory Inc). The following primary antibodies were used: Guinea pig anti-AaegVasa ([39], 1:3 k), rabbit anti-cleaved-Caspase 3 (Cell Signaling Technology, #9661,1:200,), mouse anti-H2Av (DSHB, UNC93-5.2.1, 1:1000), mouse anti-PH3 (Abcam, ab14955, 1:10 k). DNA was labelled by TO-PRO-3 (Invitrogen, T3605, 1:5 k) or Hoechst 33,342 (Thermo Fisher Scientific, H21492, 1:5 k). Fluorescein-conjugated phalloidin (Thermo Fisher Scientific, F432, 1:200) was used to label the actin cytoskeleton. After staining, the testes and ovaries were mounted on glass slides and imaged using a Leica SP8 confocal microscopy system. The same image acquisition settings were used for age-matched control and irradiated samples. Images were processed with Adobe Photoshop and Adobe Illustrator.

### Sperm quantification in male mosquito testes

Sperm quantification in testes was based on [62] with modifications. In brief, 7- to 8-day unmated males, non-irradiated or irradiated, were dissected individually in 20 µL PBS on a glass slide. The sixth (VI) abdominal tergite was carefully removed from the distal end of the male mosquito's abdomen. The testes were isolated and the seminal vesicle was separated from the male accessory glands and placed onto a new glass slide. Testes and seminal vesicles were ruptured and pulled apart using a pair of insect pins and the suspension was homogenized by gentle pipetting. To dilute the sample, 180 µL PBS was added to the suspension and used to rinse the insect pins. The final sperm solution (200 µL) was homogenized by drawing the suspension through a pipette and pipetting 20 times. Five 1 µL drops of sperm suspension were transferred onto a microscope slide and then dried at room temperature. Each droplet was then fixed with 2 µL ethanol (70%), dried at room temperature, and stained with 2 µL of DAPI solution (20 µg/mL, Thermo Fisher Scientific, 62,248, 1:50) for 10 min. The number of sperm present in the testes and seminal vesicles was then manually counted under a fluorescent microscope (Nikon – Eclipse 80i). The total sperm number per adult was calculated (mean of five of 1 µL droplets × 200).

### Sperm quantification in female mosquito spermathecae

Sperm quantification in spermathecae was based on [63] with modifications. In brief, after 7-day mating, females were aspirated, knocked out on the ice, and dissected in 20 µL PBS on a glass well plate. The 3 spermathecal lobes were isolated from the distal region of the abdomen, transferred to a glass slide, ruptured with a pair of insect pins and homogenized by pipetting 20 times. Care was taken when rupturing the lobes so as not to damage the sperm while also allowing the separation of the aggregated sperm inside the lobes. Five 1 µL drops of the suspension were placed onto a microscope slide and allowed to dry completely under room temperature before fixation in 2 µL ethanol (70%) and dried again. Once dry, 2 µL of DAPI solution (20 µg/mL) was used to stain each droplet for 10 min. The slide was then viewed under a fluorescent microscope and the number of sperm in each droplet was counted. The total number of sperm present in all spermathecal lobes was then calculated (mean of the five 1 µL × 20).

### Sperm viability assay

This method was adapted from [62]. The Live/Dead Sperm Viability Kit (Thermo Fisher Scientific, L7011) was used. In brief, the seminal vesicles and testes from the irradiated males 72-h post-irradiation were dissected, transferred onto a glass slide and ruptured in 10 µL physiological saline. An additional 10 µL (total 20 µL) of physiological saline was added to the sperm solution and homogenized by pipetting thoroughly. The first dye, SYBR 14—membrane permeable dye used to identify live cells—was added at a concentration of 100 nM and the sample was incubated at 28 °C for 5 min. Propidium iodide, a membrane-impermeable stain for dead or dying cells, was added to the sample at a final concentration of 12 µM and incubated at 28 °C for another 5 min. One 2 µL droplet was placed on a microscope slide and covered immediately with a glass coverslip (22 × 22 mm), the edges were sealed to reduce drying. Slides were observed using an upright fluorescence microscope (Zeiss Imager M2 Upright) at × 20 magnification. A GFP/FITC filter (ex: 470/40 em: 525/50) was used to image SYBR 14 stained nuclei and Cy3 filter (ex 560/55 em: 645/75) was used to image propidium iodide-stained cell membranes. Three 2 µL droplets from each sample were scanned and the total number was used to quantify the sperm viability. Images were taken with a Leica SP8 confocal microscope system.

### Statistical analyses

Statistical analyses were performed using GraphPad Prism software. The Kruskal–Wallis test was employed to compare the egg number and hatching rate, followed by Dunn’s multiple comparison test as a post hoc analysis. Kaplan-Meyer tests were used to estimate the median survival time of adult mosquitoes during longevity experiments followed by pairwise comparisons of survival curves that were performed by the Log-rank (Mantel-Cox) test. Comparisons of the average PH3-positive or cleaved-Caspase3-positive somatic cells per ovariole, sperm number, and sperm viability were performed by unpaired *t*-test (two-tailed). Comparisons of the percentage of PH3-positive or cleave-Caspase3-positive testis percentages in the tests were performed by Fisher’s exact test (two-tailed).

### Supplementary Information

**Additional file 1.** X-ray irradiation causes limited apoptosis in testes. Representative confocal microscopy images of testes with Vasa (Green), Cleaved-Caspase3 (Red), and DNA (Grey) staining at Day 0 (A), Day 1 (C), Day 3 (E), Day 7 (G), and Day 14 (I) in controls, and at Day 0 (B), Day 1 (D), Day 3 (F), Day 7 (H) and Day 14 (J) after irradiation. (K) Percentage of Cleaved-Caspase3 -positive testes. Comparisons of control and irradiated samples are performed by Fisher's Exact Test; *** (*P* < 0.001), ** (*P* < 0.01), ns (not significant). The number of samples in each group is shown above the X-axis.

**Additional file 2.** Pupae packing for X-ray irradiation. Top view (A) and lateral view (B) of a petri dish (94x16mm) with 20 mL water and 200 pupae (100 male and 100 female pupae).

**Additional file 3.** Schematic drawing of the X-ray chamber in the Radsource RS2400V X-ray irradiator.

**Additional file 4. **Schematic drawing of the placement of ionization chamber and acrylic holder inside the X-ray canister of RS2400V X-ray irradiator during dose rate measurement. 

**Additional file 5.** Schematic drawing of petri dishes and Gafchromic^TM^ file setup for dose rate and dose uniformity measurement.

**Additional file 6.** Dose mapping and dose uniformity of effective pupae irradiating area within the X-ray canister of RS2400V irradiator showing the dose distribution relative to the mean dose.

## Data Availability

All data generated or analysed during this study are included in this published article and its supplemental information files.

## Abbreviations

Ae. Aegypti: Aedes aegypti
D. melanogaster: Drosophila melanogaster
CI: Cytoplasmic incompatibility
GSC: Germline stem cell
IGS: Inner germarium sheath cell
IIT: Incompatible insect technique
PH3: Phosphorylated Histone H3 (pSer10)
SIT: Sterile insect technique
