# Supplementary figures and images for: Different mechanisms of X-ray irradiation-induced male and female sterility in Aedes aegypti
Source: BMC Biol. 2023 Nov 27;21:274. doi: 10.1186/s12915-023-01757-1 (PMC10683188; doi:10.1186/s12915-023-01757-1)

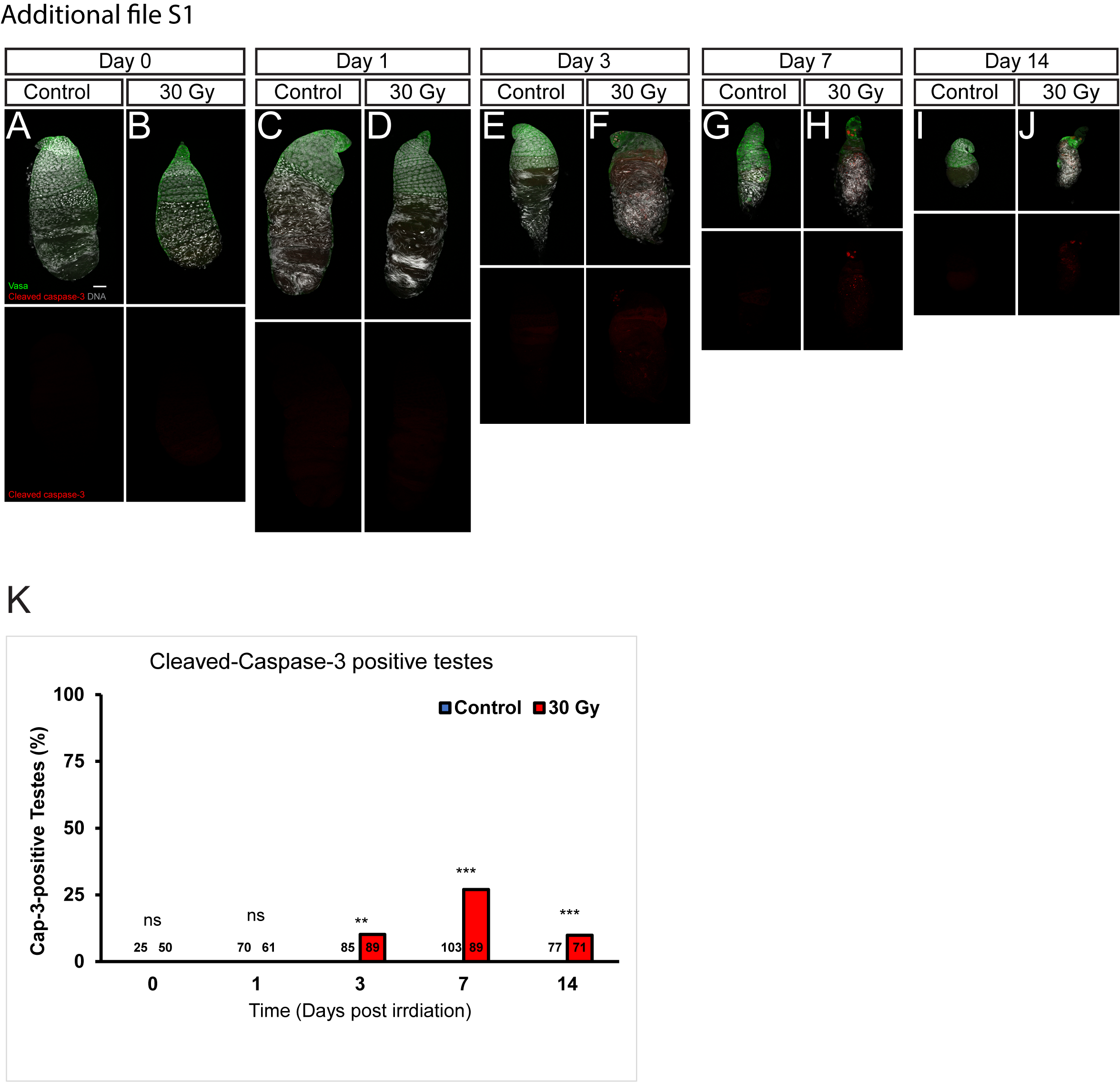

Supplement: Supplementary file 1 — Additional file 1. X-ray irradiation causes limited apoptosis in testes. Representative confocal microscopy images of testes with Vasa (Green), Cleaved-Caspase3 (Red), and DNA (Grey) staining at Day 0 (A), Day 1 (C), Day 3 (E), Day 7 (G), and Day 14 (I) in controls, and at Day 0 (B), Day 1 (D), Day 3 (F), Day 7 (H) and Day 14 (J) after irradiation. (K) Percentage of Cleaved-Caspase3 -positive testes. Comparisons of control and irradiated samples are performed by Fisher's Exact Test; *** (P < 0.001), ** (P < 0.01), ns (not significant). The number of samples in each group is shown above the X-axis. [file 12915_2023_1757_MOESM1_ESM.tif]

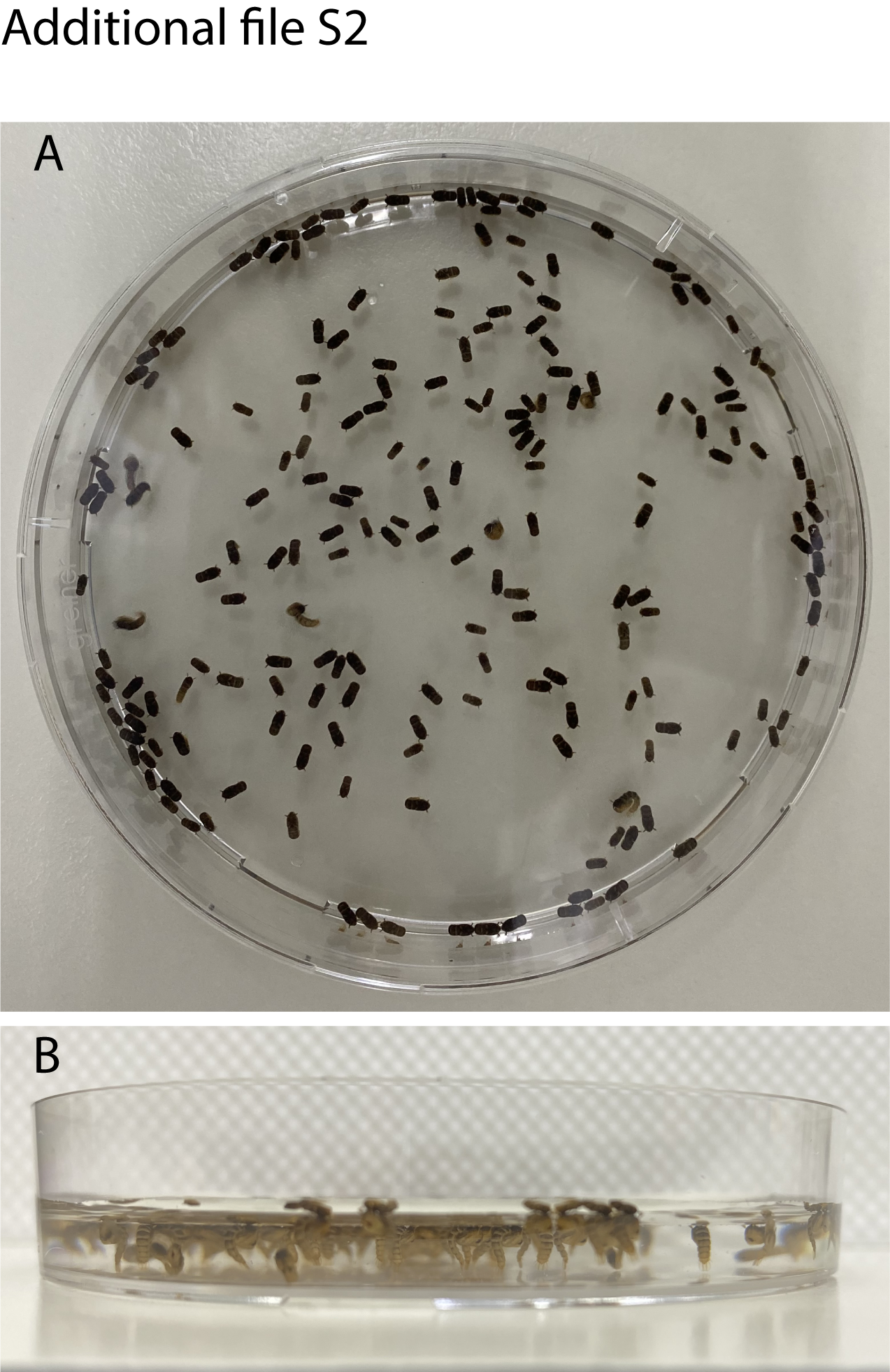

Supplement: Supplementary file 2 — Additional file 2. Pupae packing for X-ray irradiation. Top view (A) and lateral view (B) of a petri dish (94x16mm) with 20 mL water and 200 pupae (100 male and 100 female pupae). [file 12915_2023_1757_MOESM2_ESM.tif]

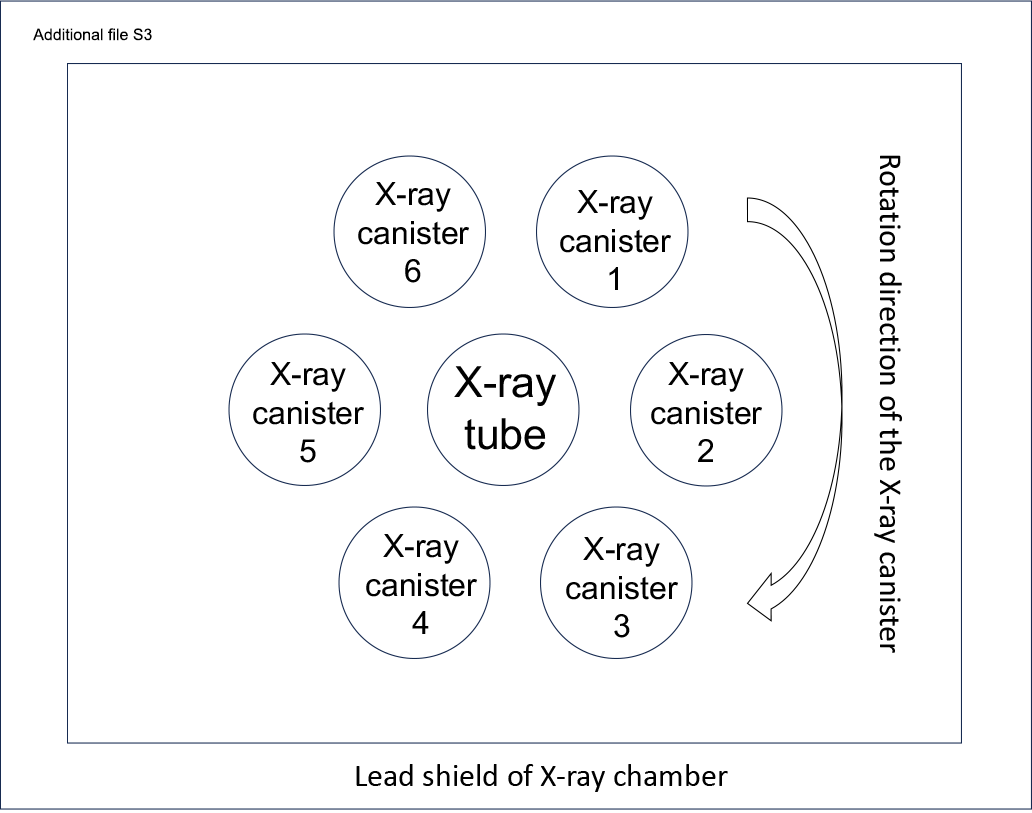

Supplement: Supplementary file 3 — Additional file 3. Schematic drawing of the X-ray chamber in the Radsource RS2400V X-ray irradiator. [file 12915_2023_1757_MOESM3_ESM.tif]

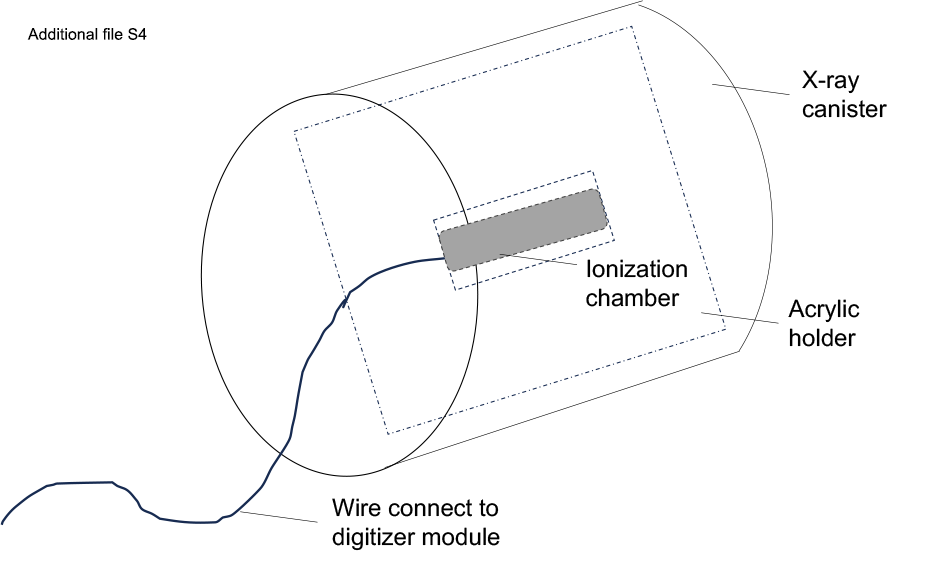

Supplement: Supplementary file 4 — Additional file 4. Schematic drawing of the placement of ionization chamber and acrylic holder inside the X-ray canister of RS2400V X-ray irradiator during dose rate measurement. [file 12915_2023_1757_MOESM4_ESM.tif]

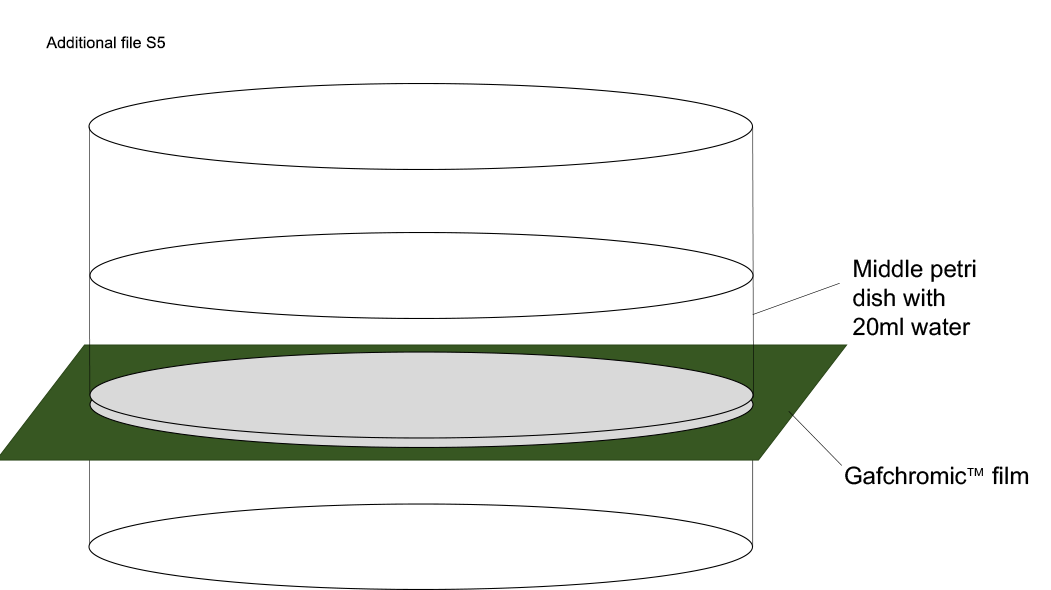

Supplement: Supplementary file 5 — Additional file 5. Schematic drawing of petri dishes and GafchromicTM file setup for dose rate and dose uniformity measurement. [file 12915_2023_1757_MOESM5_ESM.tif]

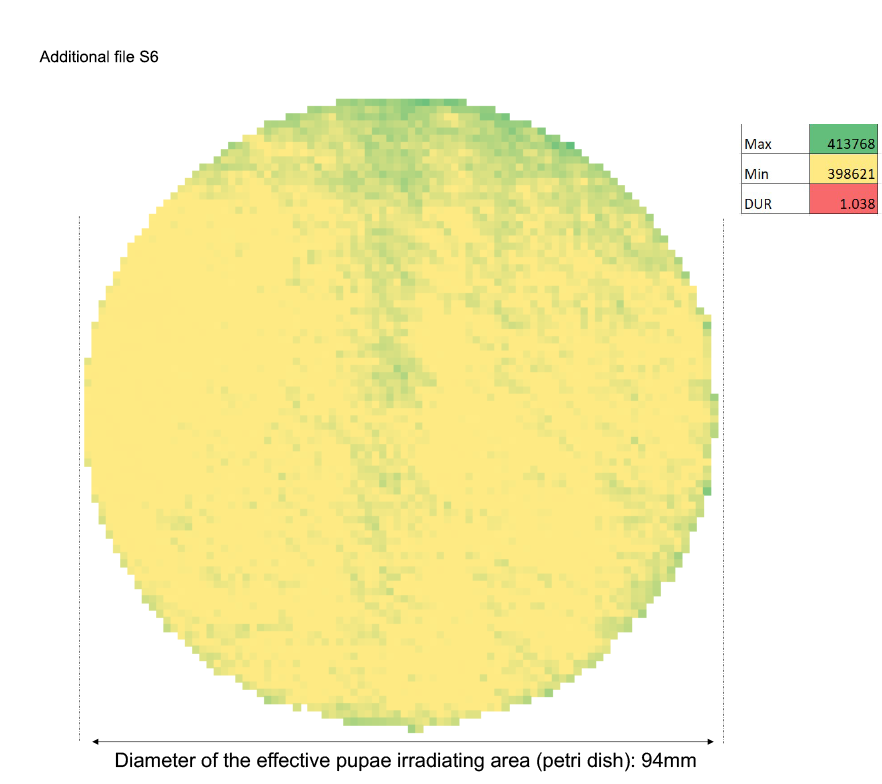

Supplement: Supplementary file 6 — Additional file 6. Dose mapping and dose uniformity of effective pupae irradiating area within the X-ray canister of RS2400V irradiator showing the dose distribution relative to the mean dose. [file 12915_2023_1757_MOESM6_ESM.tif]
